# Zinc supplementation and light intensity affect 2-acetyl-1-pyrroline (2AP) formation in fragrant rice

**DOI:** 10.1186/s12870-022-03954-6

**Published:** 2023-04-11

**Authors:** Jiang Shuochen, Zhang Lihe, Hu Fenqin, Tang Xiangru, Du Bin

**Affiliations:** 1 College of Agriculture, South China Agricultural University, Guangzhou, 510642 Guangdong China; 2 Agricultural Genomics Institute at Shenzhen, Chinese Academy of Agricultural Sciences, Shenzhen, 434007 Guangdong China

**Keywords:** Shading, Zinc application level, Aroma synthesis precursors, Aroma synthesis related enzymes, Antioxidase

## Abstract

**Background:**

Improving the yield and aroma content of fragrant rice is the focus of fragrant rice research. Light and Zinc (Zn) management generally cause regulations in the 2-acetyl-1-pyrroline (2AP) accumulation in fragrant rice. In addition, Zn promotes rice growth and improves rice yield, which has the potential to compensate for the negative impact of low light on fragrant rice yield. However, the potential of Zn to improve fragrant rice yield and 2AP content under shading conditions has not been verified.

**Methods:**

Field experiments were conducted in the rice season (May–September) in 2019 to 2021. Two light i.e., normal light (NL) and low light (LL) and four Zn levels i.e., 0 kg Zn ha^− 1^ (N0), 1 kg Zn ha^− 1^ (Zn1), 2 kg Zn ha^− 1^(Zn2), and 3 kg Zn ha^− 1^ (Zn3), which applied at booting stage was set up. The grain yield, 2AP contents, Zn content in polished rice, photosynthesis related indicators, MDA content, antioxidant enzyme activity and the biochemical parameters related to 2AP formation were investigated.

**Results:**

Shading reduced yield by 8.74% and increased 2AP content by 24.37%. In addition, shading reduced net photosynthetic rate (Pn), superoxide dismutase (SOD), peroxidase (POD) and catalase (CAT), and increased proline, γ-aminobutyric acid (GABA), and pyrroline-5-carboxylic acid (P5C), proline dehydrogenase (PDH), △1-pyrroline-5-carboxylic acid synthetase (P5CS), malondialdehyde (MDA). With increasing Zn application levels, yield, 2AP, Zn content in polished rice, Pn, proline, P5C, GABA, PDH, P5CS, SOD, CAT and POD increased, and MDA decreased. Significant Light and Zn interaction effect on 2AP content was detected, and both shading and increasing Zn application increased the 2AP content.

**Conclusion:**

Shading can increase the 2AP content but reduce the yield of fragrant rice. Increasing Zn application under shading conditions can further promote the biosynthesis of 2AP, but the effect of improving yield is limited.

**Supplementary Information:**

The online version contains supplementary material available at 10.1186/s12870-022-03954-6.

## Introduction

Rice is an extremely important food crop for human beings [8]. Fragrant rice is widely loved by people because of its strong aroma, excellent taste and rich nutrition, so it has attracted the attention of researchers in rice-producing countries [7]. Aroma is an important indicator of the value-added of fragrant rice, so aroma characteristic substances and their formation mechanisms have been paid attention to in the research of fragrant rice [21, 22]. Previous studies have found that there are more than 100 kinds of aroma volatile substances in fragrant rice, among which 2-acetyl-1-pyrroline (2AP) is the most important characteristic of fragrant rice aroma [2, 3, 17]. The content of 2AP is not only an important substance to distinguish aromatic rice from non-aromatic rice, but also the main reason for the difference in aroma between aromatic rice varieties [19, 29, 42]. Therefore, the aroma of aromatic rice and their regulation of formation have attracted more and more attention in the research on aroma enhancement of aromatic rice.

There have been numerous reports related to the synthesis of 2AP, and it was found that proline, ornithine, glutamate, Δ1-pyrroline, γ-aminobutyraldehyde, Δ1-pyrroline-5-carboxylic acid (P5C), acetyl groups are important precursors involved in the synthesis of 2AP [36, 41, 54]. In addition, the enzymes such as proline dehydrogenase (PDH, EC 1.5.99.8), Δ1-pyrroline-5-carboxylate synthase (P5CS, EC 2.7.2.11/1.2.1.41), ornithine aminotransferase (OAT, EC 2.6.1.13), diamine oxidase and (DAO, EC 1.4.3.22) were reported to be the crucial enzymes involved in 2AP biosynthesis in fragrant rice [54, 57, 59]. Studying the prerequisite substances and enzyme activities related to aroma synthesis is conducive to a deeper understanding of the mechanism of aroma formation.

Light is one of the most important environmental factors affecting rice growth, development and productivity [24]. Shading stress caused by environmental pollution, climate change and crowded plants reduce light intensity, which leads to changes in the morphology, physiology, biomass, grain yield, and quality of rice [56]. Xie et al. [53, 55] reported that shading decline grain filling rate, 1000-grain weight, biomass, harvest index, nitrogen (N) uptake and yield. Li et al. [18, 20] reported that shading significantly reduced grain yield of rice primarily by affecting the grain-filling progress after the heading stage. Overall, shading restricts photosynthetic capacity, interferes disturbance of accumulation, partitioning, and remobilization of dry matter and carbohydrates, and finally decrease the grain yield [18, 20, 38, 52, 53, 55]. However, Xie et al. [54] reported that shading decreased grain yield, but increased 2AP content and selectively increased the precursors and enzymes activities involved in the synthesis of 2AP. Therefore, it is important to enhance the growth and yield of fragrant rice with improved quality characters under low light conditions.

Zinc (Zn) is a component of many enzymes in plants and plays an important role in protein synthesis, carbohydrate metabolism, cell division, and gene expression [45]. Numerous literatures have reported that Zn can improve the antioxidant capacity and photosynthesis capacity of rice, improve crop grain yield, rice quality and zinc content in rice [31, 47, 58]. Perhaps increasing Zn application is an effective way to make up for the loss of rice photosynthesis capacity caused by shading. In addition, increasing Zn application rate also promoted the synthesis of 2AP in fragrant rice [4, 21, 22, 27, 28]. However, there are few reports on the yield and 2AP content of fragrant rice under different Zn application rate under shading conditions.

The present study was aimed to investigate the effects of Zn application rate on grain yield, 2AP contents as well as physio-biochemical attributes of fragrant rice under shading conditions. We hypothesized that increasing Zn supply can compensate for the negative impact of shading on photosynthetic capacity and further increase 2AP content.

## Material and methods

### Experimental site

Experiments were conducted in 2019 to 2021 at the Yangtze University farm, Jingzhou County, Hubei Province, China (112°04′N-112°05 N, 30°32′E-30°33′E). The area belongs to the northern subtropical agricultural climate zone, with annual average temperature of 16.5 °C (Nearly 20 years, 2000–2020), annual average precipitation of 1095 mm, and annual average sunshine time of 1718 h. In the experimental field, soil texture is clay loam, and the index of soil agrochemical properties were 6.09 of pH, 29.78 g kg^− 1^ of organic matter, 224.56 mg kg^− 1^ of available N, 11.03 mg kg^− 1^ of available P, 124.35 mg kg^− 1^ of available K, 0.58 mg kg^− 1^ of available Zn. Fragrant rice cultivar was Xiangyaxiangzhan which are well-known and widely cultivated fragrant rice cultivars in China and favored by consumers due to their special aroma and enchant flavor.

### Experimental treatments and design

The experiment was arranged in split-plot design with Zn application levels as the main plots and light treatments as the subplots. In the study, there were four Zn application levels and two light treatment, each treatment was designed with three replications and totally 24 subplots (4.0 m × 6.0 m) were established in the field.

Light treatment included normal light (NL) and low light (LL). The light treatment was conducted according to the method of Mo et al. [33], low light was a shading level equivalent to 67% reduction of full natural light, and shading during the whole phase of grain filling (30 days). Four Zn levels were 0 kg Zn ha^− 1^ (Zn0), 1 kg Zn ha^− 1^ (Zn1), 2 kg Zn ha^− 1^(Zn2), and 3 kg Zn ha^− 1^ (Zn3), which foliar applied at booting stage in the form of zinc chloride. All the treatments received N (90 kg ha^− 1^), P_2_O_5_ (59 kg ha^− 1^) and K_2_O (120 kg ha^− 1^) in the form of urea, calcium super phosphate and potassium chloride at basal, respectively. Then an additional dose of N (45 kg N ha^− 1^) was applied to all the treatment at tillering stage. In the field, plastic film was covered on all soil ridges and installed to a depth of 25 cm below soil surface for minimizing seepage between plots. Rice was sown on May 1 and transplanted on June 1 in the 3 years. After 30 days of sowing, seedlings at three-leaf stage were transplanted with 2 seedlings per hill at 20 × 20 cm planting distance. Other field management, such as weeds, pests, and diseases etc. were intensively controlled in order to avoid yield loss.

### Measurement items and methods

#### Yield

Grain yields were measured at maturity by taking 5 m^2^ plant samples at the center of each plot. The filled grains in each 5 m^2^ plant sample were separated from the straws. The filled grains and straws were oven-dried at 70 °C to a stable weight and weighed, and grain yield was calculated at 14% moisture content.

#### Determination of zinc content in polished rice

The Zn contents of polished rice were measured with an atomic absorption spectrophotometer (Perkin Elmer, CA, USA) [43]. The processing of polished rice samples before testing were oven dried, ground, digested in an acid mixture (HClO_4_ + HNO_3_; 3:10 ratio) on a digestion plate.

#### Leaf area index and net photosynthetic rate

Five planting pits of rice plants were selected in each plot to measure the leaf area index (LAI) and net photosynthetic rate (Pn) at 15 days after grain filling. LAI was obtained by an area of fully-expanded leaves on the plants divided by rice planting area [16]. The net photosynthetic rate of the top fully-expanded leaves on the main-stem were determined by gas exchange analyzer (Li-6400, Li-COR Inc., NE, USA) between 9:30–11:00 am when the photosynthetic active radiation above the canopy was 1200 mmol m^− 2^ s^− 1^.

#### Determination of 2-acetyl-1-pyrroline content in grain

At maturity stage, the fresh grain sample from 1 hill with 5 replicates for each treatment were harvested and frozen by liquid N_2_ and immediately stored at − 20 °C. Grain samples were estimated for 2AP content by synchronization distillation and extraction method combined with GCMS-QP 2010 Plus (Shimadzu Corporation, Japan) as described by Mo et al. [33].

#### Determination of proline, γ-aminobutyric acid (GABA), and pyrroline-5-carboxylic acid (P5C) contents in leaves

At maturity stage, the fresh leaf sample from 1 hill with 5 replicates for each treatment were harvested and frozen by liquid N_2_ and immediately stored at − 80 °C for biochemical analyses. The contents of proline, GABA and P5C were assayed by following the methods of Xie et al. [53–55]. Specifically, the proline contents were determined by using ninhydrin, and the absorbance was read at 520 nm. The final proline contents were expressed as μg/g fresh weight (FW) of leaves. The GABA contents were measured follow by: plant tissue was grinded with 2.5 mL of 60% ethanol, then placed in the oscillation instrument (HZS-H, China) for 4 h and then 1 mL of 60 mM lanthanum chloride was added, shaken for 15 min and centrifuged at 2000 g for 5 min. The supernatant was then added with 0.125 mL of 1 M KOH and centrifuged at 2000 g for 5 min again. The supernatant (0.4 mL) was mixed with 0.12 mL of borate buffer (0.2 M, pH 10.0), 0.4 mL of 6% phenol solution, and 80 μL of sodium hypochloride (10% available chlorine), kept in the boiling water for 10 min, and then placed in ice water for 5 min. Later, 0.4 mL of 60% ethanol was added to the reaction mixture and the absorbance was recorded at 645 nm. The GABA content was calculated by comparing with the standard curve and expressed as mg g^− 1^ FW. The reaction mixture for determination of P5C content contained 0.2 ml supernatant of enzyme extract, 0.5 ml of 10% trichloroacetic acid (TCA), and 0.2 ml of 40 mM 2-aminobenzaldehyde. The absorbance was read at 440 nm, and the contents were expressed as μmol g^− 1^.

#### Determination of the activities of proline dehydrogenase (PDH), △1-pyrroline-5-carboxylic acid synthetase (P5CS), Ornithine Aminotransferase (OAT), and Diamine Oxidase (DAO)

The PDH, P5CS, OAT and DAO activities were assayed by following the methods of Xie et al. [53, 55]. Specifically, The absorbance after reaction was read for determination of PDH activity, and the reaction mixture contained L-proline (15 mM), cytochrome c (0.01 mM), phosphate buffer (100 m M, pH 7.4), 0.5% (v/v) Triton X-100, and the enzyme extract (0.1 ml) in a total volume of 0.5 ml. The reaction mixture was incubated at 37 °C for 30 min, and the reaction was terminated by adding 0.5 ml of 10% trichloroacetic acid (TCA). After adding 0.5 ml of 0.5% 2-aminobenzaldehyde in 95% ethanol, the mixture was further incubated at 37 °C for 10 min and centrifuged at 8000 rpm for 10 min, and the absorbance of the supernatant was read at 440 nm, the absorbance change of 0.1 in 1 min was defined as one unit of enzyme activity, and the activity was expressed as U g^− 1^ FW.

The reaction mixture for determination of P5CS activity included 50 mM Tris-HCL buffer, 20 mM MgCl_2_, 50 mM sodium glutamate, 10 mM ATP, 100 mM hydroxamate-HCL, and 0.5 ml of enzyme extract. The reaction was started by the addition of 0.5 ml of enzymatic extracts. After 5 min at 37 °C, the reaction was stopped by addition of 0.5 ml of a stop buffer (2.5% of FeCl_3_ plus 6% of trichloroacetic acid, dissolved in 100 ml of 2.5 M HCl). The absorbance after the reaction was read at 440 nm, the absorbance change of 0.1 in 1 min was defined as one unit of enzyme activity, and the activity was expressed as U g^− 1^ FW.

The reaction medium for determination of OAT activity contained 100 mM potassium phosphate buffer pH 8.0, 50 mM ornithine, 20 mM α-ketoglutarate, 1 mM pyridoxal 5-phosphate, and the enzyme extract (0.1 ml)—the final total volume was 1 ml. The reaction medium was incubated at 37 °C for 30 min. The reaction was stopped by adding 0.5 ml trichloroacetic acid (10%), and the color was developed by incubating the reaction mixture with 0.5 ml o-amino benzaldehyde (0.25%) in ethanol (95%) for 1 hr. After centrifugation at 8000 rpm for 10 min, the clear supernatant fraction was taken to measure the absorbance at 440 nm. The absorbance change of 0.1 in 1 min was defined as one unit of enzyme activity; the activity was expressed as U g^− 1^ FW.

The reaction solutions for determination of DAO activity contained 2.5 ml 0.1 M sodium phosphate buffer (pH 6.5), 0.1 ml crude enzyme extracts, 0.1 ml peroxidase (250 U ml^− 1^), and 0.2 ml 4-aminoantipyrine/ N, N-dimethylaniline reaction solutions. The reaction was initiated by the addition of 0.1 ml 20 mM Put. A 0.01 value of the changes in absorbance at 440 nm was regarded as one activity unit of the enzyme, and the activity was expressed as U g^− 1^ FW.

#### Determination of antioxidase activities and malondialdehyde content

The fresh flag leaf samples were grinded with liquid nitrogen to form homogenate, 9 ml of 50 mM sodium phosphate buffer (pH 7.8) was added to the homogenate, and then centrifuged at 8000 rpm for 15 min at 4 °C. The supernatant was gathered for determination of superoxide dismutase (SOD), peroxidase (POD), catalase (CAT) enzyme activities as well as malondialdehyde (MDA) contents referring to Huang et al. [14]. One unit of SOD activity was the amount of enzyme that induced 50% inhibition in the initial rate of reduction of nitroblue tetrazolium at 560 nm. The mixture for determining POD consists of 1 ml of sodium phosphate buffer (pH 7.8), 0.95 ml of 0.2% guaiacol, 1 ml of 0.3% H_2_O_2_ and 0.05 ml aliquot of enzyme extract. The absorbance was read at 470 nm for 90 s with an interval of 30 s. One unit of POD activity was defined as the amount of enzyme that caused the decomposition of 1 mg substrate at 470 nm. The mixture for determining CAT consists of 1 ml of 1.95 ml distilled water, 1 ml of 0.3% H_2_O_2_ and 0.05 ml enzyme extract. The absorbance was read at 470 nm for 90 s with an interval of 30 s. One unit of CAT activity was the decomposition of 1 M H_2_O_2_ at A240 within 1 min in 1 g of fresh leaves samples. The 1.5 ml enzyme extract was used to determine the content of MDA. The enzyme extract was mixed with 0.5 ml thiobarbituric acid solution prepared in 5% trichloroacetic acid and boiled at 100 °C for 30 min. The samples after cooling were centrifuged at 3000 rpm for 15 min. The absorbance was read at 450 nm, 532 nm and 600 nm. The MDA contents were computed with the formula: MDA content = 6.45(OD_532_ − OD_600_) − 0.599OD_450_.

### Statistical analyses

All experimental data were collected in 2019 to 2021 and expressed as mean ± standard error (SE) of three replicates. The normal distribution and homogeneity variance of data were tested using Shapiro-Wilk’s test and Levene’s test on SPSS 21.0, respectively. Differences of indicators between 3 years was compared by one-way analysis of variance. Multi-factor analysis of variance was used for revealing the effects of Zn application levels, shading treatments, and interaction among them respectively. In statistic analysis, 2 significance levels were set at *p* < 0.05 and *p* < 0.01. The Spearman’s correlation coefficients (r values) were determined to evaluate the relationships between various characteristics of plant. The histograms were drawn by Origin, and the heat map was drawn by TBtools.

## Results

### Variance analysis

Except for LAI, other rice indicators were significantly different in 2019–2021 (Table 1). Differences in weather between 3 years cause year to has a significant impact on rice indicators (Supplementary Fig. 1). Except for the activities of Zn content, LAI, OAT and DAO, light treatment had significant effects on other indicators of rice. Except for the activities of LAI, OAT and DAO, Zn treatment had significant effects on other indicators of rice. The interaction of light treatment and Zn treatment had a significant effect on 2AP content, proline, P5C, GABA, PDH, P5CS, MDA, SOD, CAT and POD.

**Table 1:** The ANOVA for the effect of year (Y), light (L) and zinc (Zn) on rice indicator. Asterisks indicate significant difference at different levels (*P < 0.05, **P < 0.01, ***P < 0.001)

	Yield	2AP	Zn content	Pn	LAI	Proline	P5C	GABA	ProDH	P5CS	OAT	DAO	MDA	SOD	CAT	POD
Year	**	*	***	**	ns	***	*	***	***	**	***	***	***	*	*	*
Light	***	***	ns	***	ns	***	***	***	***	***	ns	ns	***	***	***	***
Zinc	***	***	***	***	ns	***	***	***	***	***	ns	ns	***	***	***	***
Y*L	ns	ns	ns	ns	ns	ns	ns	ns	ns	ns	ns	ns	ns	ns	ns	ns
Y*N	ns	ns	ns	ns	ns	ns	ns	ns	ns	ns	ns	ns	ns	ns	ns	ns
L*N	ns	**	ns	ns	ns	***	***	*	**	*	ns	ns	***	***	**	*
Y*L*N	ns	ns	ns	ns	ns	ns	ns	ns	ns	ns	ns	ns	ns	ns	ns	ns

### Yield, 2AP content and Zn content in polished rice

**Fig. 1:**
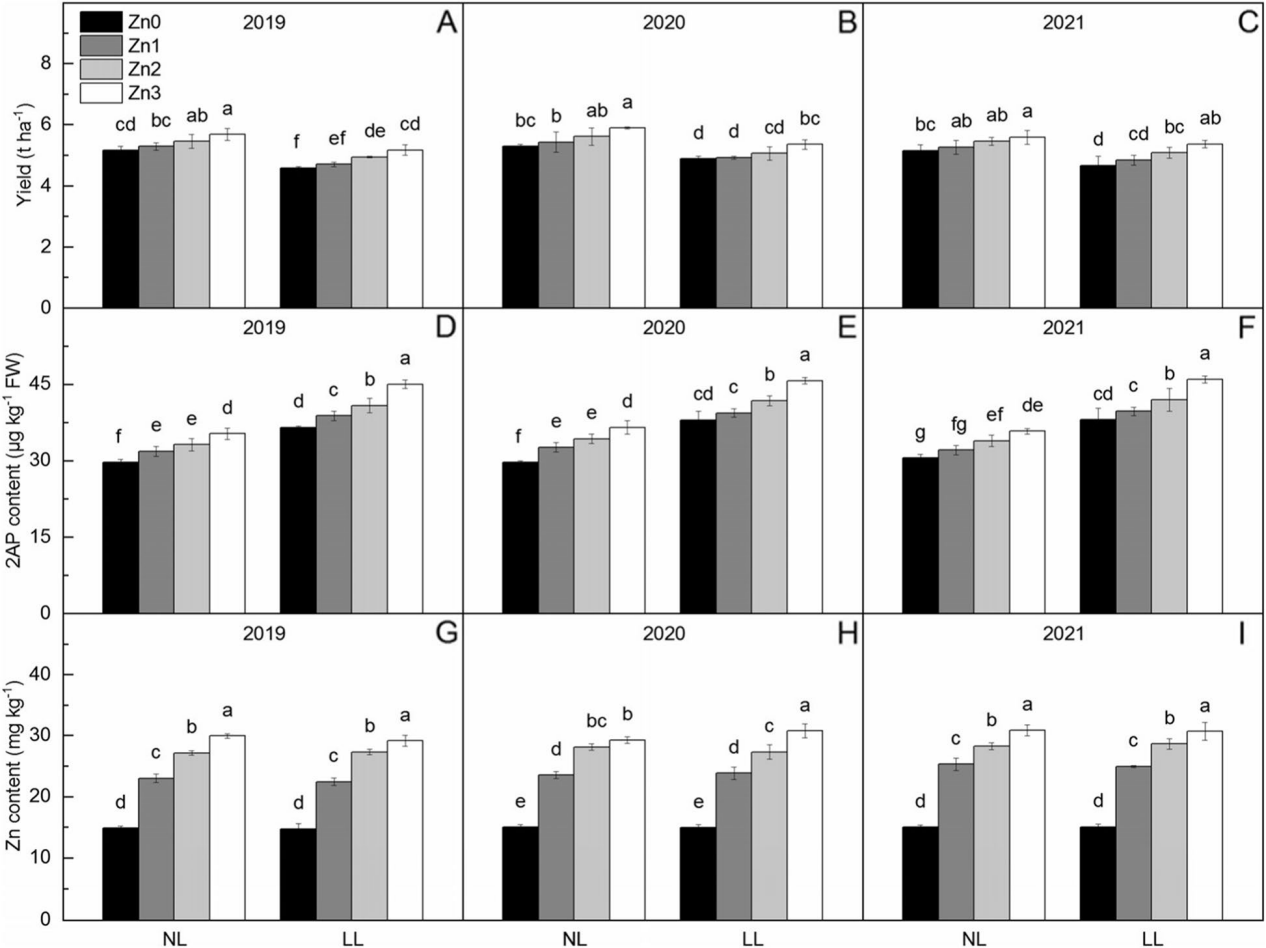
Yield, 2AP content in grain and Zn content in polished rice under two light treatments and four Zn application levels in 2019 to 2021. Zn0, Zn1, Zn2 and Zn3 mean 0 kg Zn ha^−1^, 1 kg Zn ha^−1^, 2 kg Zn ha^−1^, and 3 kg Zn ha^−1^ applied at booting stage, respectively. NL and LL mean normal light and low light, respectively. Different lowercase letters indicate statistical differences among treatments at *P* < 0.05

Shading significantly reduces yield by 8.74% and increases 2AP content by 24.37% (Fig. 1). Yield, 2AP content and Zn content in polished rice increased with Zn application, and compared with Zn0, Zn application increased yield by 2.47% ~ 11.25%, increased 2AP by 5.91% ~ 20.70% and increased Zn content in polished rice by 59.93% ~ 101.90%, respectively. From Zn1 to Zn3, the increase of 2AP under normal light were lower than that under low light. It shows that increasing Zn application level under shading conditions more effectively improve 2AP content.

### Pn and LAI

**Fig. 2:**
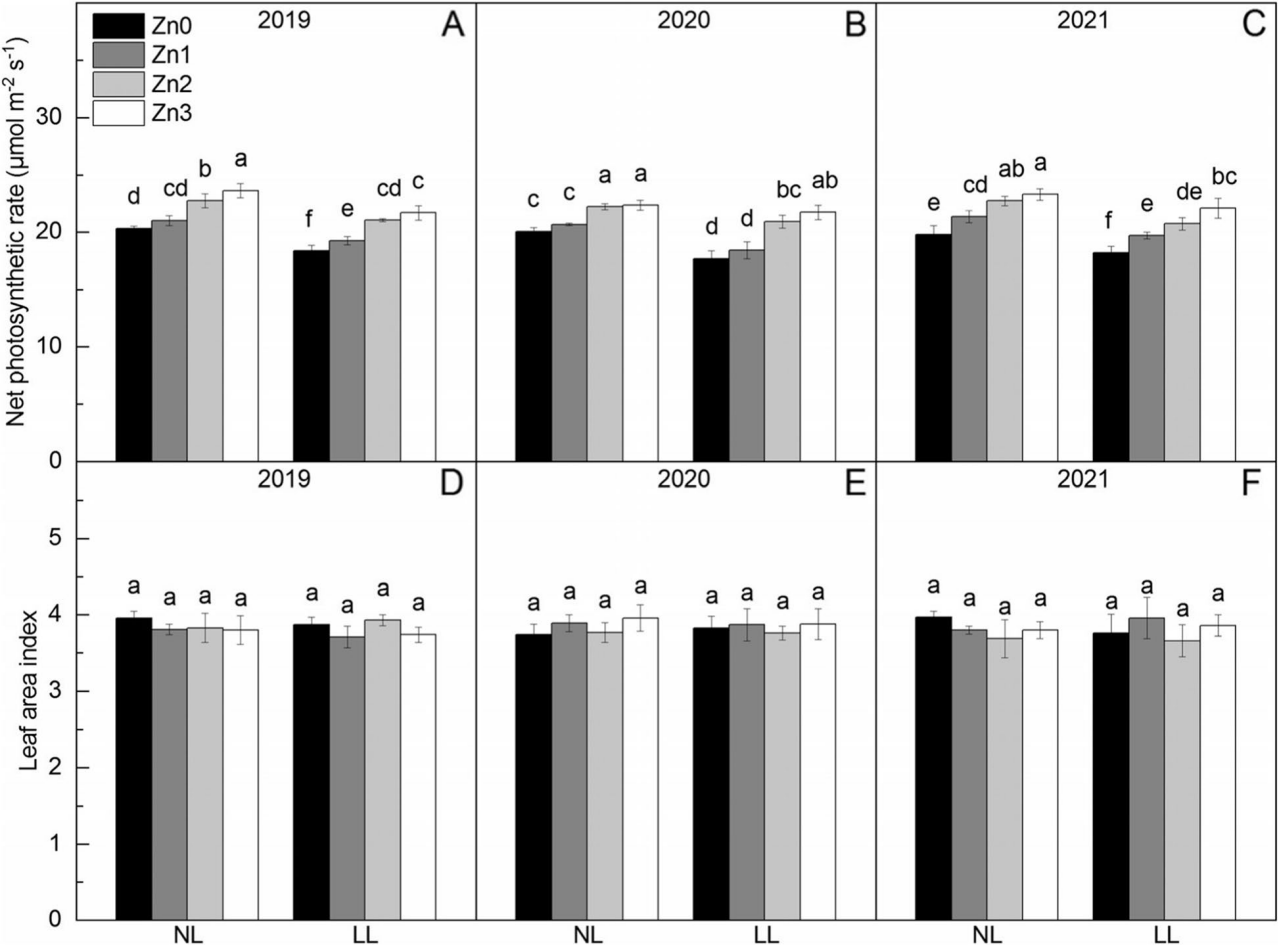
Net photosynthetic rate and leaf area index content of rice under two light treatments and four Zn application levels in 2019 to 2021. Zn0, Zn1, Zn2 and Zn3 mean 0 kg Zn ha^− 1^, 1 kg Zn ha^− 1^, 2 kg Zn ha^− 1^, and 3 kg Zn ha^− 1^ applied at booting stage, respectively. NL and LL mean normal light and low light, respectively. Different lowercase letters indicate statistical differences among treatments at *P* < 0.05

Shading significantly reduces Pn by 7.82% (Fig. 2). Pn increased significantly with increasing Zn application level, and compared with Zn0, Zn application increased the Pn by 5.22% ~ 17.78%.

### Contents of proline, P5C and GABA

**Fig. 3:**
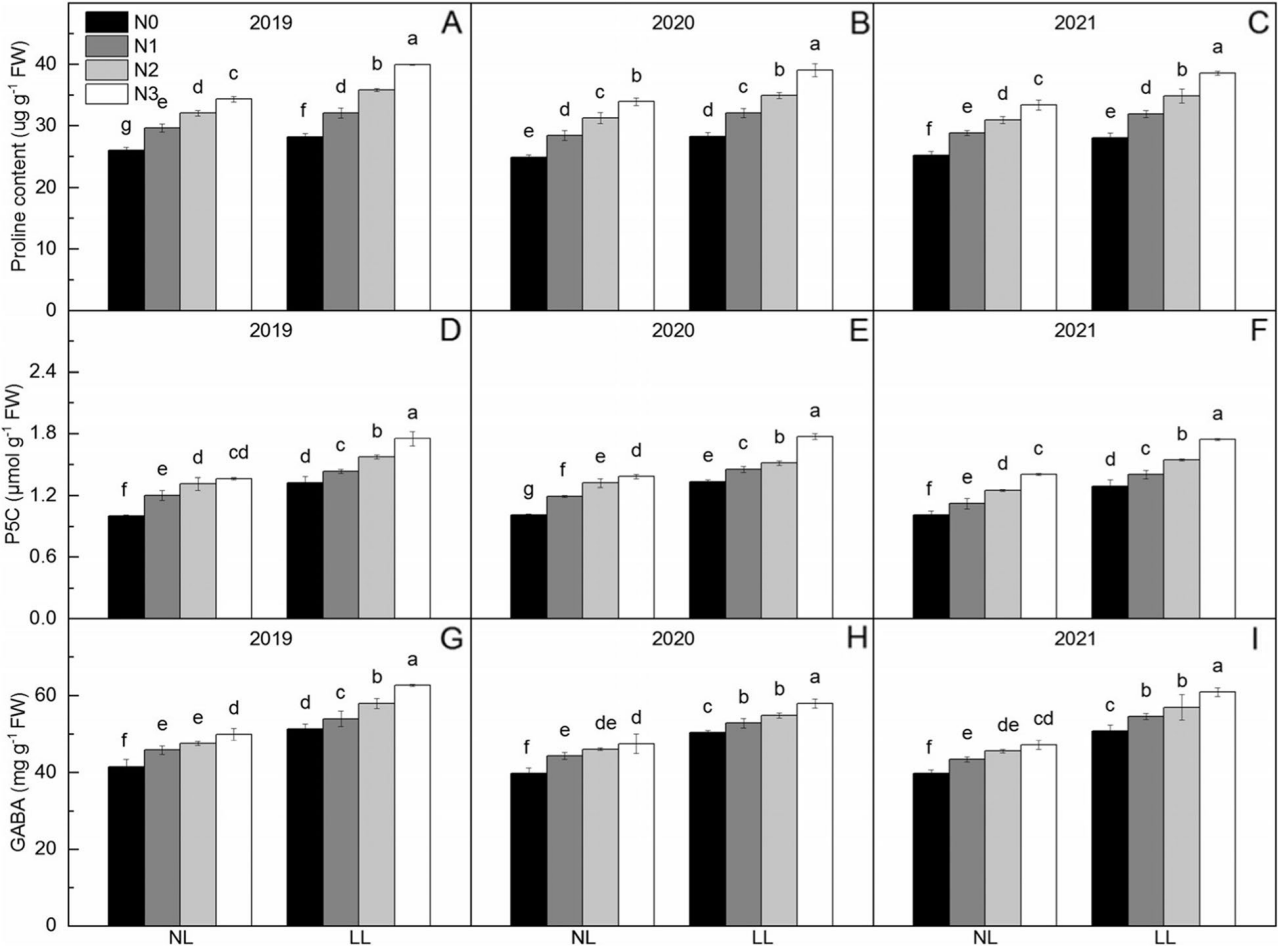
Proline, γ-aminobutyric acid (GABA), and pyrroline-5-carboxylic acid (P5C) contents in leaves of rice under two light treatments and four Zn application levels in 2019 to 2021. Zn0, Zn1, Zn2 and Zn3 mean 0 kg Zn ha^− 1^, 1 kg Zn ha^− 1^, 2 kg Zn ha^− 1^, and 3 kg Zn ha^− 1^ applied at booting stage, respectively. NL and LL mean normal light and low light, respectively. Different lowercase letters indicate statistical differences among treatments at *P* < 0.05

Shading significantly increased the contents of proline, P5C and GABA by 12.53, 24.20, and 23.61%, respectively (Fig. 3). The contents of proline, P5C and GABA increased significantly with increasing Zn application level, and compared with Zn0, Zn application increased proline content by 14.03% ~ 36.60%, increased by P5C content by 12.18% ~ 35.3.%, and increased by 7.85% ~ 19.26%, respectively. From Zn1 to Zn3, the increase of proline, P5C and GABA under normal light were lower than that under low light.

### Activities of PDH, P5CS, OAT and DAO

**Fig. 4:**
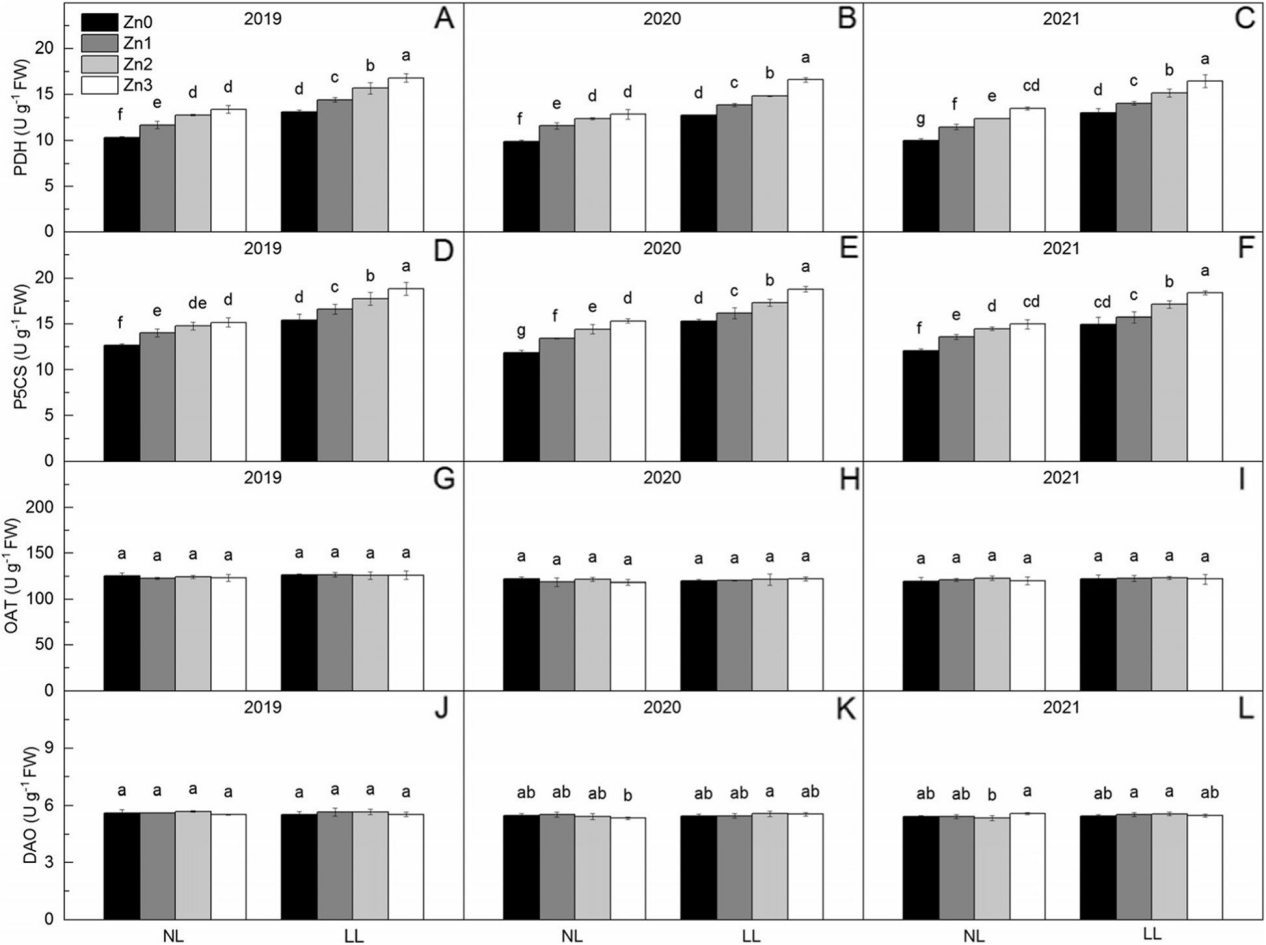
Proline dehydrogenase (PDH), △1-pyrroline-5-carboxylic acid synthetase (P5CS), ornithine aminotransferase (OAT), and diamine oxidase (DAO) in leaves of rice under two light treatments and four Zn application levels in 2019 to 2021. Zn0, Zn1, Zn2 and Zn3 mean 0 kg Zn ha^− 1^, 1 kg Zn ha^− 1^, 2 kg Zn ha^− 1^, and 3 kg Zn ha^− 1^ applied at booting stage, respectively. NL and LL mean normal light and low light, respectively. Different lowercase letters indicate statistical differences among treatments at *P* < 0.05

Shading significantly increased the activities of PDH and P5CS by 24.49 and 21.46%, respectively (Fig. 4). The activities of PDH and P5CS increased with Zn application. Compared with Zn0, Zn application increased PDH activity by 11.66% ~ 29.82% and increased P5CS activity by 8.82% ~ 23.47%, respectively. From Zn1 to Zn3, increase of activities of PDH and P5CS under normal light waere lower than that under low light.

### MDA content and activities of SOD, CAT and POD

**Fig. 5:**
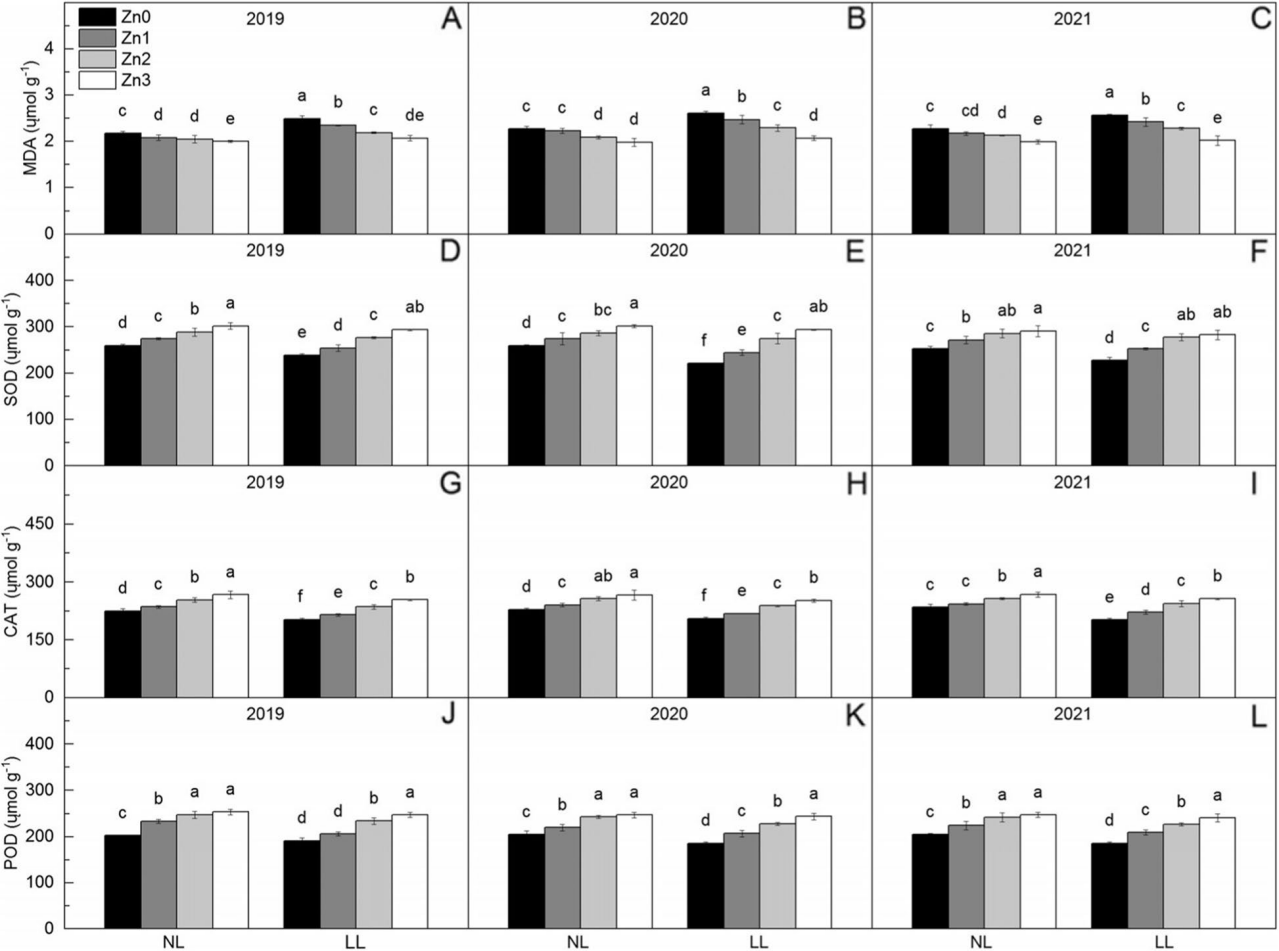
Activities of superoxide dismutase (SOD), peroxidase (POD), catalase (CAT) and malondialdehyde (MDA) content in leaves of rice under two light treatments and four Zn application levels in 2019 to 2021. Zn0, Zn1, Zn2 and Zn3 mean 0 kg Zn ha^− 1^, 1 kg Zn ha^− 1^, 2 kg Zn ha^− 1^, and 3 kg Zn ha^− 1^ applied at booting stage, respectively. NL and LL mean normal light and low light, respectively. Different lowercase letters indicate statistical differences among treatments at *P* < 0.05

Shading significantly rose MDA content by 9.49% and declined the activities of SOD, CAT and POD by 5.94% ~ 7.68% (Fig. 5). The MDA content decreased and the activities of SOD, CAT and POD rose with Zn application. Compared with Zn0, Zn application decreased MDA content by 4.54% ~ 15.60%, increased SOD activity by 7.67% ~ 20.94%, increased CAT activity by 5.87% ~ 20.37% and increased POD activity by 10.50% ~ 25.76%, respectively. From Zn1 to Zn3, decrease of MDA content and increase of SOD, CAT and POD under normal light waere lower than that under low light.

### Correlation analysis

**Fig. 6:**
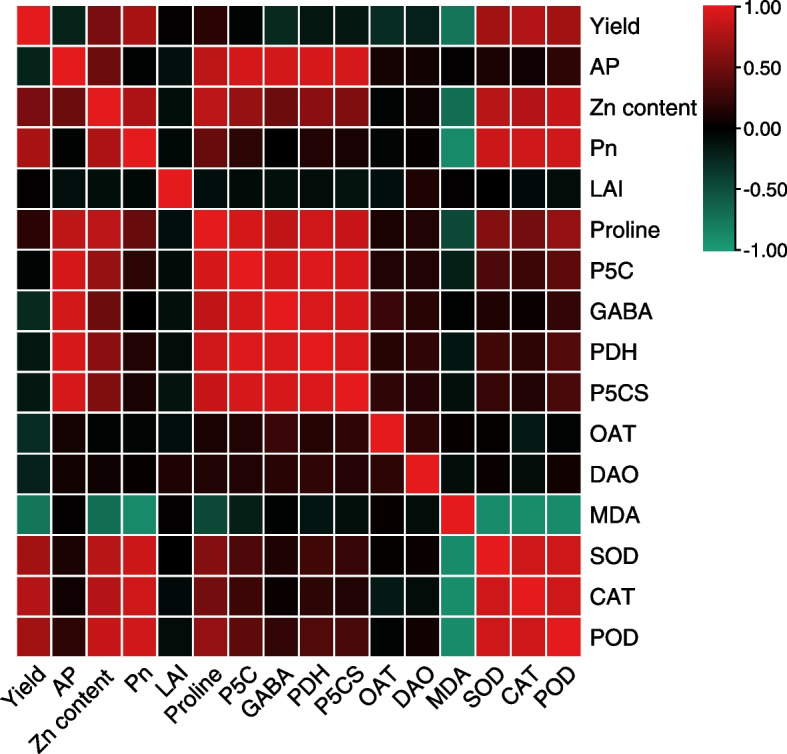
Correlation analysis among rice indicators

Yield was significantly positively correlated with Zn content in polished rice, Pn, SOD, CAT and POD, and significantly negatively correlated with GABA, OAT and MDA (Fig. 6). 2AP content was significantly positively correlated with Zn content in polished rice, proline, P5C, GABA, PDH, P5CS. Zn content in polished rice was significantly positively correlated with Pn, proline, P5C, GABA, PDH, P5CS, SOD, CAT and POD, and significantly negatively correlated MDA. Pn was significantly positively correlated with proline, SOD, CAT and POD, and significantly negatively correlated MDA. In addition, positive associations existed among the proline, P5C, GABA, PDH and P5CS, and also existed among the SOD, CAT and POD. Proline was significantly negatively correlated with MDA, and proline, P5C, PDH, P5CS were significantly positively correlated with SOD and POD, and proline and P5C were significantly positively correlated with CAT.

## Discussion

Although fragrant rice has higher taste quality and price due to its aroma, the low grain yield is not conducive to ensuring the security of grain quantity, which is the main obstacle to the development of fragrant rice industry [54]. In this study, shading reduced yield and net photosynthetic rate, which was consistent with previous conclusions [38, 53, 55]. Shading reduces light intensity, resulting in decreased photosynthetic capacity, limiting the absorption and distribution of dry matter, and ultimately reducing yield [52]. As a component of more than 300 enzymes, Zn is involved in the metabolic process of carbohydrates, lipids, proteins, nucleic acids and other important substances, and plays an important role in the growth and development of plants [45]. In this study, yield, net photosynthetic rate and Zn accumulation in polished rice were significantly improved with increasing Zn application, which was consistent with previous conclusions [11, 40]. Zn regulate biochemical reactions in the photosynthetic metabolism by integrating the structure of Rubisco and rising β-carbonic anhydrase activity and chlorophyll concentration to improve crop photosynthetic capacity and yield [44, 50]. With the increase of Zn application levels, the yield increase under shading treatment did not increase significantly compared with normal light. This indicated that the effect of supplementing the negative effect of shading on the yield of fragrant rice by increasing zinc fertilizer was limited. Improving the yield of fragrant rice under shade requires optimizing Zn application strategies or exploring more effective ways to increase yield.

Zn is one of the essential micronutrients and has multiple roles in the human body including the efficient functioning of cellular metabolic activities and stimulation of the immune system [34]. Rice is the staple food for more than 50% of the world’s population [8]. It is an economical, efficient and safe way to supplement the Zn intake of residents by increasing the concentration and bioavailability of Zn in polished rice [45, 49]. In this study, the Zn content in polished rice increased with the increase of Zn application, which is consistent with the previous conclusion [30], indicating that foliar application of Zn can also increase the Zn content in aromatic rice milled rice. It is worth noting that the concentration of Zn in polished rice has exceeded the concentration that is beneficial to humans (24 mg· kg^− 1^) in Zn2 [32]. Therefore, foliar spraying of Zn with 2 kg ha^− 1^ is a suitable concentration to increase the Zn concentration in polished rice.

Previous research has shown that 2AP is the primary component responsible for the fragrant rice aroma [36]. 2AP is synthesized by the following pathways: firstly, proline, glutamate and ornithine are catalyzed by PDH, P5CS and OAT, respectively, into P5C and then 2AP is directly formed with an acetyl group or methylglyoxal (MG) [27, 28, 51]. Secondly, ornithine is catalyzed by ODC into putrescine, then putrescine catalyzed by DAO into GABald and further forms Δ1-pyrroline via the non-functional BADH2 enzyme and 2AP is ultimately synthesized from Δ1-pyrroline and an acetyl group or MG [4, 42]. Previous studies have reported that the environment affects 2AP biosynthesis in aromatic rice, including light and Zn [4, 6, 18, 20, 27, 28, 33]. We explored the effects of light and Zn treatments and their interactions on 2AP biosynthesis, providing more evidence for the effects of light and zinc on 2AP content. In our study, shading increased 2AP, proline, P5C and GABA contents consistent with the report of Mo et al. [33]. Proline and P5C are precursors for 2AP synthesis [25–28]. Ortiz-Alcaide et al. [37] found that shading enhanced the activity of abscisic acid (ABA), and [12] reported that ABA can induce the synthesis of P5C. Furthermore, ABA accumulation in plants has been determined to regulate expression of P5CS gene [1]. Similarly, Cao et al. [5] reported that exogenous ABA significantly upregulated the expression of OsP5CS1, OsP5CS2, and OsProDH. We found that shading also increased the activity of P5CS and PDH. It shows that shading can promote the biosynthesis of 2AP by increasing the activity of P5CS and PDH to promote the synthesis of P5C and proline. The process may be accompanied by an increase in ABA, but it is necessary to determine the ABA content and synthetic substances to further prove. Shading will increase the content of glutamate, which is catalyzed by GAD into GABA [46], which may be the reason why shading increases GABA content.

Bao et al. [4] reported that Zn application increased 2AP, proline, P5C, P5CS and PDH, but had no significant effect on GABA. Luo et al. [27, 28] reported that Zn application increased 2AP, P5C, proline, PDH, P5CS and DAO, and the magnitude of the increase in GABA varied with cultivars. In this study, 2AP, proline, P5C, GABA content, P5CS and PDH activities increased with increasing Zn application. The same view of previous report and our study is that Zn application increases 2AP content through glutamate pathway and proline pathway. We found that Zn increased GABA content, possibly because Zn increased glutamate content, promoting GAD to catalyze glutamate to GABA. Nie et al. [35] reported that Zn may promote glutamate synthesis by increasing GOGAT activity and GDH activity supporting our results. When plants lack N, arginine in plants will be decomposed into ornithine and urea to provide N sources for plants [39], in which the accumulation of ornithine upregulates the activity of ODC and DAO that converts ornithine to GABald [13]. Differences in cultivar, environment, and N application strategy lead to differences in N metabolism in rice that account for the differences between our study and the study of Luo et al. Shading and Zn application are both effective ways to increase aroma content of fragrant rice, and the content of 2AP is the highest under shading and foliar spraying of Zn with 3 kg· ha^− 1^.

In plants, MDA is an important indicator of oxidative stress, while SOD, POD and CAT are important antioxidant enzymes [15]. In our study, shading increased MDA content and decreased the activities of three antioxidant enzymes. Reportedly, the effects of shading on the antioxidant system of rice varied by cultivar, and in cultivars tolerant to low-light stress, shading increases antioxidant capacity and reduces MDA content [23]. Shading reduced the antioxidant capacity of Xiangyaxiangzhan, indicating that it may be a cultivar that cannot tolerate low light stress. Therefore, it is our next research direction to screen fragrant rice varieties that are resistant to low light stress and study their more physiological characteristics under low light.

The activities of three antioxidant enzymes increased and MDA content decreased with increasing Zn application. Similarly, Song et al. [48] reported that foliar spraying of ZnO nanoparticles reduced MDA content, increased antioxidant enzyme activity, and ameliorated low temperature-induced oxidative stress in rice. Faizan et al. [9, 10] reported that foliar spraying of ZnO nanoparticles enhanced rice antioxidant enzyme activity, decreased hydrogen peroxide and MDA content, and ameliorated cadmium- and copper-induced oxidative stress. Previous studies supported our view that Zn could enhance the antioxidant capacity of fragrant rice. In addition, under shading conditions, Zn was more effective in increasing the activity of antioxidant enzymes and reducing the content of MDA, indicating that Zn could improve the oxidative stress induced by shading in Xiangyaxiangzhan.

Mo et al. [33] reported that shading increased 2AP content but decreased yield, and when only light was treated, there was a negative correlation between 2AP content and yield. In this study, there was no significant correlation between yield and 2AP content. The reason may be that yield and 2AP content increased with the increase of Zn application. This was also demonstrated by the significant positive correlation between Zn content in polished rice and yield and 2AP content. This verify that increasing Zn application has the potential to simultaneously increase yield and 2AP content. 2AP content was significantly positively correlated with proline, P5C, GABA, PDH, P5CS, which was consistent with the previous conclusion [25, 26, 54]. The results of correlation analysis showed that Zn increased yield by increasing Pn and antioxidant enzyme activity and reducing MDA content, which was consistent with the previous conclusion [9–11]. In addition, Zn increased 2AP content by increasing contents of proline, P5C and GABA, and activities of PDH and P5CS.

## Conclusion

Shading at grain filling stage reduced yield but increased 2AP content. Increased Zn application at booting stage improved yield and 2AP content. Increased Zn application under shading conditions can further improve 2AP content, but yield increase is limited. Therefore, our next research direction is to optimize the Zn application strategy to improve the compensation potential of Zn for the negative impact of shading on yield.

## Supplementary Information

**Additional file 1: Supplementary Fig. 1.** Maximum temperature, minimum temperature, mean temperature, daily rainfall and daily sunshine hours from April 1 to September 15 in the experiment conducted during 2019–2021.

## Data Availability

The datasets used and/or analysed during the current study are available from the corresponding author on reasonable request.

## Abbreviations

2AP: 2-acetyl-1-pyrroline
P5C: Δ1-pyrroline-5-carboxylic acid
Zn: Zinc
PDH: Proline dehydrogenase
P5CS: Δ1-pyrroline-5-carboxylate synthase
OAT: Ornithine aminotransferase
DAO: Diamine oxidase
Pn: Net photosynthetic rate
NL: Normal light
LL: Low light
LAI: Leaf area index
GABA: γ-aminobutyric acid
SOD: Superoxide dismutase
POD: Peroxidase
CAT: Catalase
MDA: Malondialdehyde
MG: Methylglyoxal

## References

[1] Ashraf M, Foolad MR (2007). Roles of glycine betaine and proline in improving plant abiotic stress resistance. Environ Exp Bot.

[2] Ashraf U, Hussain S, Akbar N, Anjum SA, Hassan W, Tang X (2018). Water management regimes alter Pb uptake and translocation in fragrant rice. Ecotoxicol Environ Saf.

[3] Ashraf U, Mahmood MH, Hussain S, Abbas F, Anjum SA, Tang X (2020). Lead (Pb) distribution and accumulation in different plant parts and its associations with grain Pb contents in fragrant rice. Chemosphere.

[4] Bao G (2021). Transcriptomic analysis provides insights into foliar zinc application induced Upregulation in 2-Acetyl-1-pyrroline and related transcriptional regulatory mechanism in fragrant Rice. J Agric Food Chem.

[5] Cao X, Wu L, Wu M, Zhu C, Jin Q, Zhang J (2020). Abscisic acid mediated proline biosynthesis and antioxidant ability in roots of two different rice genotypes under hypoxic stress Bmc. Plant Biol.

[6] Chen J (2021). Light quality during booting stage modulates fragrance, grain yield and quality in fragrant rice. J Plant Interact.

[7] Custodio MC, Cuevas RP, Ynion J, Laborte AG, Velasco ML, Demont M (2019). Rice quality: how is it defined by consumers, industry, food scientists, and geneticists?. Trends Food Sci Technol.

[8] Deng N, Grassini P, Yang H, Huang J, Cassman KG, Peng S (2019). Closing yield gaps for rice self-sufficiency in China. Nat Commun.

[9] Faizan M, Bhat JA, Hessini K, Yu F, Ahmad P (2021). Zinc oxide nanoparticles alleviates the adverse effects of cadmium stress on *Oryza sativa* via modulation of the photosynthesis and antioxidant defense system. Ecotox Environ Safe.

[10] Faizan M, Bhat JA, Noureldeen A, Ahmad P, Yu F (2021). Zinc oxide nanoparticles and 24-epibrassinolide alleviates cu toxicity in tomato by regulating ROS scavenging, stomatal movement and photosynthesis. Ecotox Environ Safe.

[11] Farooq M (2018). Application of zinc improves the productivity and biofortification of fine grain aromatic rice grown in dry seeded and puddled transplanted production systems. Field Crop Res.

[12] Ghosh UK, Islam MN, Siddiqui MN, Cao X, Khan M (2022). Proline, a multifaceted signalling molecule in plant responses to abiotic stress: understanding the physiological mechanisms. Plant Biol (Stuttg).

[13] González-Hernández AI, Scalschi L, Vicedo B, Marcos-Barbero EL, Morcuende R, Camañes G (2022). Putrescine: a key metabolite involved in plant development, Tolerance and Resistance Responses to Stress. Int J Mol Sci.

[14] Huang S (2020). Application of inorganic passivators reduced Cd contents in brown rice in oilseed rape-rice rotation under Cd contaminated soil. Chemosphere (Oxford).

[15] Jiang S, Du B, Wu Q, Zhang H, Deng Y, Tang X, et al. Selenium decreases the cadmium content in brown rice: foliar Se application to plants grown in Cd-contaminated soil. J Soil Sci Plant Nutr. 2022;22(1):1033-43.

[16] Jiang S, Du B, Wu Q, Zhang H, Zhu J. Increasing pit-planting density of rice varieties with different panicle types to improves sink characteristics and rice yield under alternate wetting and drying irrigation. Food Energy Secur. 2021. 10.1002/fes3.335.

[17] Kanu AS, Ashraf U, Mo Z, Sabir SU, Baggie I, Charley CS, Tang X (2019). Calcium amendment improved the performance of fragrant rice and reduced metal uptake under cadmium toxicity. Environ Sci Pollut Res Int.

[18] Li QP (2020). Shading decreases rice yield by impeding grain-filling progress after heading. Agron J.

[19] Li Y (2019). γ-Aminobutyric acid regulates grain yield formation in different fragrant Rice genotypes under different nitrogen levels. J Plant Growth Regul.

[20] Li Y (2020). Light and water treatment during the early grain filling stage regulates yield and aroma formation in aromatic rice. Sci Rep.

[21] Li Y (2021). ZnO nanoparticle-based seed priming modulates early growth and enhances physio-biochemical and metabolic profiles of fragrant rice against cadmium toxicity. J Nanobiotechnology.

[22] Li Y, Liang L, Huang S, Li W, Ashraf U, Ma L, Mo Z (2021). Exogenous Melatonin and Catechol Application Modulate Physio-Biochemical Attributes and Early Growth of Fragrant Rice Under Cd Toxicity. J Soil Sci Plant Nutr.

[23] Liu Q, Wu X, Chen B, Ma J, Gao J (2014). Effects of low light on agronomic and physiological characteristics of Rice including grain yield and quality. Rice Sci.

[24] Lu X, Zhou Y, Fan F, Peng J, Zhang J (2020). Coordination of light, circadian clock with temperature: the potential mechanisms regulating chilling tolerance in rice. J Integr Plant Biol.

[25] Luo H (2020). Biofortification with chelating selenium in fragrant rice: effects on photosynthetic rates, aroma, grain quality and yield formation. Field Crop Res.

[26] Luo H (2020). Exogenous proline induces regulation in 2-acetyl-1-pyrroline (2AP) biosynthesis and quality characters in fragrant rice (Oryza sativa L.). Sci Rep-Uk.

[27] Luo H, Du B, He L, He J, Hu L, Pan S, Tang X (2019). Exogenous application of zinc (Zn) at the heading stage regulates 2-acetyl-1-pyrroline (2AP) biosynthesis in different fragrant rice genotypes. Sci Rep-Uk.

[28] Luo H, Du B, He L, Zheng A, Pan S, Tang X (2019). Foliar application of sodium selenate induces regulation in yield formation, grain quality characters and 2-acetyl-1-pyrroline biosynthesis in fragrant rice. BMC Plant Biol.

[29] Ma L (2021). Application of hydrogen-rich water modulates physio-biochemical functions and early growth of fragrant rice under cd and Pb stress. Environ Sci Pollut Res Int.

[30] Mabesa RL, Impa SM, Grewal D, Johnson-Beebout SE (2013). Contrasting grain-Zn response of biofortification rice (Oryza sativa L.) breeding lines to foliar Zn application. Field Crop Res.

[31] Majumder S, Datta K, Datta SK (2019). Rice biofortification: high Iron, zinc, and vitamin-a to fight against “hidden hunger”. Agronomy (Basel).

[32] Meenakshi JV (2010). How cost-effective is biofortification in combating micronutrient malnutrition? An ex ante assessment. World Dev.

[33] Mo Z, Li W, Pan S, Fitzgerald TL, Xiao F, Tang Y, et al. Shading during the grain filling period increases 2-acetyl-1-pyrroline content in fragrant rice. Rice. 2015;8:1-10. 10.1186/s12284-015-0040-y PMC4384914 25844114

[34] Nakandalage N, Nicolas M, Norton RM, Hirotsu N, Milham PJ, Seneweera S (2016). Improving Rice zinc biofortification success rates through genetic and crop management approaches in a changing environment front. Plant Sci.

[35] Nie Z, Wang J, Rengel Z, Liu H, Gao W, Zhao P (2018). Effects of nitrogen combined with zinc application on glutamate, glutamine, aspartate and asparagine accumulation in two winter wheat cultivars. Plant Physiol Biochem.

[36] Okpala NE, Mo Z, Duan M, Tang X (2019). The genetics and biosynthesis of 2-acetyl-1-pyrroline in fragrant rice. Plant Physiol Biochem.

[37] Ortiz-Alcaide M, Llamas E, Gomez-Cadenas A, Nagatani A, Martinez-Garcia JF, Rodriguez-Concepcion M (2019). Chloroplasts modulate elongation responses to canopy shade by retrograde pathways involving HY5 and Abscisic acid. Plant Cell.

[38] Pan S (2016). Effects of nitrogen and shading on root morphologies, nutrient accumulation, and photosynthetic parameters in different rice genotypes. Sci Rep-Uk.

[39] Patel J, Ariyaratne M, Ahmed S, Ge L, Phuntumart V, Kalinoski A, Morris PF (2017). Dual functioning of plant arginases provides a third route for putrescine synthesis. Plant Sci.

[40] Pooniya V, Shivay YS, Rana A, Nain L, Prasanna R (2012). Enhancing soil nutrient dynamics and productivity of basmati rice through residue incorporation and zinc fertilization. Eur J Agron.

[41] Poonlaphdecha J (2016). Biosynthesis of 2-acetyl-1-pyrroline in rice calli cultures: demonstration of 1-pyrroline as a limiting substrate. Food Chem.

[42] Poonlaphdecha J, Maraval I, Roques S, Audebert A, Boulanger R, Bry X, Gunata Z (2012). Effect of timing and duration of salt treatment during growth of a fragrant rice variety on yield and 2-acetyl-1-pyrroline, proline, and GABA levels. J Agric Food Chem.

[43] Prasad R (2006). Zinc in soils and in plant, human & animal nutrition. Indian J Fertilisers.

[44] Qiao X, He Y, Wang Z, Li X, Zhang K, Zeng H (2013). Effect of foliar spray of zinc on chloroplast β-carbonic anhydrase expression and enzyme activity in rice (Oryza sativa L.) leaves. Acta Physiol Plant.

[45] Rehman H, Aziz T, Farooq M, Wakeel A, Rengel Z, Cakmak I, Hoffland E (2012). Zinc nutrition in rice production systems: a review. Plant Soil.

[46] Shelp BJ, Bozzo GG, Trobacher CP, Zarei A, Deyman KL, Brikis CJ (2012). Hypothesis/review: contribution of putrescine to 4-aminobutyrate (GABA) production in response to abiotic stress. Plant Sci.

[47] Soltani SM, Hanafi MM, Samsuri AW, Muhammed SKS, Hakim MA (2016). Rice growth improvement and grains bio-fortification through lime and zinc application in zinc deficit tropical acid sulphate soils. Chem Speciat Bioavailab.

[48] Song Y, Jiang M, Zhang H, Li R. Zinc oxide nanoparticles alleviate chilling stress in Rice (Oryza Sativa L.) by regulating Antioxidative system and chilling response transcription factors. Molecules. 2021;26. 10.3390/molecules26082196. 10.3390/molecules26082196 PMC8069548 33920363

[49] Swamy B (2016). Advances in breeding for high grain Zinc in Rice. Rice (N Y).

[50] Tsonev T, Cebola Lidon FJ. Zinc in plants-an overview. Emir J Food Agric (EJFA). 2012;24(4).

[51] Wakte K, Zanan R, Hinge V, Khandagale K, Nadaf A, Henry R (2017). Thirty-three years of 2-acetyl-1-pyrroline, a principal basmati aroma compound in scented rice (Oryza sativa L.): a status review. J Sci Food Agric.

[52] Wang L, Deng F, Ren W (2015). Shading tolerance in rice is related to better light harvesting and use efficiency and grain filling rate during grain filling period. Field Crop Res.

[53] Xie W (2019). Application of gamma-aminobutyric acid (GABA) and nitrogen regulates aroma biochemistry in fragrant rice food. Sci Nutr.

[54] Xie W (2021). Application of γ-aminobutyric acid under low light conditions: effects on yield, aroma, element status, and physiological attributes of fragrant rice. Ecotox Environ Safe.

[55] Xie X, Shan S, Wang Y, Cao F, Chen J, Huang M, Zou Y (2019). Dense planting with reducing nitrogen rate increased grain yield and nitrogen use efficiency in two hybrid rice varieties across two light conditions. Field Crop Res.

[56] Yang B, Tang J, Yu Z, Khare T, Srivastav A, Datir S, Kumar V (2019). Light stress responses and prospects for engineering light stress tolerance in crop plants. J Plant Growth Regul.

[57] Yin J (2019). Silicon enhances the salt tolerance of cucumber through increasing polyamine accumulation and decreasing oxidative damage. Ecotoxicol Environ Saf.

[58] Zhang H, Wang R, Chen Z, Cui P, Lu H, Yang Y, Zhang H (2021). The effect of zinc oxide nanoparticles for enhancing Rice (Oryza sativa L.). Yield Qual Agricult (Basel).

[59] Zhu Y (2020). Silicon confers cucumber resistance to salinity stress through regulation of proline and cytokinins. Plant Physiol Biochem.

