# Supplementary material for: Zinc supplementation and light intensity affect 2-acetyl-1-pyrroline (2AP) formation in fragrant rice
Source: BMC Plant Biol. 2023 Apr 11;23:194. doi: 10.1186/s12870-022-03954-6 (PMC10088174; doi:10.1186/s12870-022-03954-6)
Supplement: Supplementary file 1 — Additional file 1: Supplementary Fig. 1. Maximum temperature, minimum temperature, mean temperature, daily rainfall and daily sunshine hours from April 1 to September 15 in the experiment conducted during 2019–2021. [file 12870_2022_3954_MOESM1_ESM.docx]

Supplementary Figure 1. Maximum temperature, minimum temperature, mean temperature, daily rainfall and daily sunshine hours from April 1 to September 15 in the experiment conducted during 2019-2021.
